# The mucosal released‐coronally advanced flap: A novel surgical approach—A case report

**DOI:** 10.1002/cap.10293

**Published:** 2024-06-10

**Authors:** Andrea Pilloni, Fabiola Dell'Olmo

**Affiliations:** ^1^ Department of Oral and Maxillofacial Sciences Section of Periodontics Sapienza University of Rome Rome Italy

**Keywords:** case report, gingival recession/surgery, oral mucosa, treatment outcome, wound healings

## Abstract

**Background:**

The coronally advanced flap (CAF) can be a predictable surgical technique for the treatment of gingival recessions. However, the characteristics of the defect (e.g., limited amount of keratinized gingiva or flap tension, etc.) may limit the use of the CAF with a possible requirement of additional surgical interventions (i.e., the use of a tissue graft to be harvested from donor sites or connective tissue substitutes).

**Methods:**

A 28‐year‐old woman patient, with no history of periodontal disease, came for referral presenting receding gums as a chief complaint, poor esthetics, and dentinal hypersensitivity at the buccal surface of teeth 11, 12, and 13. Clinically, she presented a thick phenotype with gingival recession type, RT1, with detectable cemento‐enamel junction (A‒) in the second quadrant. To reduce the need of harvesting soft tissue grafts, the amount of cutting of muscles and vessels from the inner portion of the flap and mitigate the postoperative discomfort associated with the CAF, a novel surgical approach is described here using an advanced flap that incorporates an external incision along the mucogingival junction.

**Results:**

The average root coverage achieved with the novel procedure presented in this case report was 95%, along with an increased amount of keratinized gingiva and minimal postoperative patient's discomfort.

**Conclusions:**

The mucosal released CAF is a promising technique in which the CAF technique alone may not be an indication.

**Key points:**

This technique has the following advantages:

- Reduce the need of harvesting soft tissue grafts.
- Reduce the amount of cutting of muscles and vessels from the inner portion of the flap.
- Minimal postoperative discomfort for the patient.

## INTRODUCTION

Gingival recession refers to the movement of the gingival margin (GM) toward the apex, resulting in the exposure of the root surface to the oral environment.^1^ This condition commonly occurs in populations with good oral hygiene,^2^, ^3^ particularly on the vestibular surfaces.^4^ The primary aim of any procedure addressing root coverage (RC) is to fully cover the recession defect while ensuring a pleasing appearance consistent with adjacent soft tissues and minimal probing depth (PD) postoperatively. Recently, Cairo et al.^5^ introduced a classification system for gingival recessions based on the level of interproximal clinical attachment and assessed its predictive value regarding post‐surgical RC outcomes. Three types of recession (RT) were identified: RT1, involving recession without interproximal attachment loss; RT2, with loss of attachment equal to or less than the buccal site; and RT3, showing attachment loss greater than the buccal site. This study revealed that the recession class type is strongly correlated with the extent of recession reduction postoperatively. Cairo et al. suggested that the level of interproximal attachment loss represents the highest achievable amount of RC at the buccal site post‐surgery. Notably, the RT1 class exhibited greater recession reduction compared to RT2 class, emphasizing the significance of baseline interproximal attachment loss in predicting the success of gingival recession treatment.

In existing literature,^6^, ^7^ the predictability of RC has been quantified in terms of the mean percentage of RC and the percentage of complete root coverage (CRC), reflecting the extent of soft tissue coverage over the root surface and the proportion of teeth with soft tissue margins reaching the cemento‐enamel junction (CEJ), respectively. Various surgical procedures have demonstrated success in treating gingival recessions, with RC achievable regardless of the chosen approach. However, the primary prognostic factor for post‐surgical RC remains the height of the interdental periodontal support.^8^ Periodontal probing and intra‐oral X‐rays are useful for confirming periodontal health.

The selection of a surgical technique depends on several factors, including characteristics of the defect (size and number of recession defects, presence/absence and quality of keratinized tissue (KT), dimensions of interdental soft tissue, presence of frenum or muscle pull, vestibulum depth) and patient‐related considerations.^9^ For patients prioritizing esthetics, pedicle flap techniques such as coronally advanced flap (CAF) or laterally moved flaps are recommended when sufficient KT is present around the defect. Alternatively, when KT is insufficient, pedicle flap with grafting (e.g., bilaminar technique) can improve the predictability and esthetic outcomes.^10^

Recent studies have rigorously examined anatomical and surgical factors influencing clinical outcomes. These investigations have highlighted the impact of tooth rotations, abrasions,^11^ gingival thickness,^12^ and baseline recession depth^13^ on RC outcomes. Surgical guidelines now recommend displacing the flap 2 mm or more coronally to the CEJ,^14^ ensuring passive suturing,^15^ and employing microsurgical instruments for improved outcomes.^16^ With advances in scientific understanding, CRC has become increasingly predictable, and an esthetic‐focused approach has gained prominence in plastic surgery procedures.^17^, ^18^, ^19^ Achieving CRC with soft tissue anatomy harmonious with adjacent tissues is now considered the ultimate treatment goal.^19^

Minimizing postoperative discomfort, along with satisfying esthetic requests, is extremely important when patient‐related factors are to be considered in the selection of the RC surgical approach. In fact, the CAF itself implies the use of some technical surgical steps that are the reason for postoperative sequelae (i.e., swelling, tension or pain over the area, increased bleeding). Such surgical actions are inevitable: muscle, vessels, and nerves within the thickness of the flap are severed for flap passivation. The aim of the present clinical case report is to describe a variation of the CAF technique, the mucosal released coronally advanced flap (MR‐CAF), in the treatment of RT1 multiple recessions in order to minimize the severance of important flap components and reduce patient's postoperative discomfort.

## MATERIALS AND METHODS

### Clinical presentation

A 28‐year‐old woman patient came for referral presenting receding gums as a chief complaint, poor esthetics, and dentinal hypersensitivity (DH) at the buccal surface of teeth 11, 12, and 13. Patient was a non‐smoker with no history of periodontal disease. Clinically, she presented a thick phenotype with gingival recession type RT1, with detectable CEJ (A‒) in the second quadrant. The teeth were not rotated or malpositioned and presented at least 1 mm of KT and 3–4 mm of recession depth (REC). The treatment plan included oral hygiene instructions and motivation and supra gingival scaling, followed by periodontal plastic surgery. Written informed consent was obtained from the patient.

### Case management

At baseline, the following measurements were registered by one investigator (A.P.) using a periodontal probe: REC and PD at the mid‐buccal site; KT width, the distance between the GM and the mucogingival junction (MGJ); and clinical attachment level, calculated as PD + REC. DH was recorded as present or absent (Table 1).

**TABLE 1:** Clinical data comparing pre‐surgical and post‐surgical clinical measurements at 1 year postoperatively.

	Tooth					
	11		12		13	
	Pre	Post	Pre	Post	Pre	Post
REC (mm)	3	0.5	4	1	3	0
PD (mm)	2	2	1.5	1.5	2	2
KT (mm)	1	2.5	1.5	3.5	2	4
Dentinal hypersensitivity	+	–	+	–	+	–

Abbreviations: KT, keratinized tissue; PD, probing depth; REC, recession depth.

**FIGURE 1:**
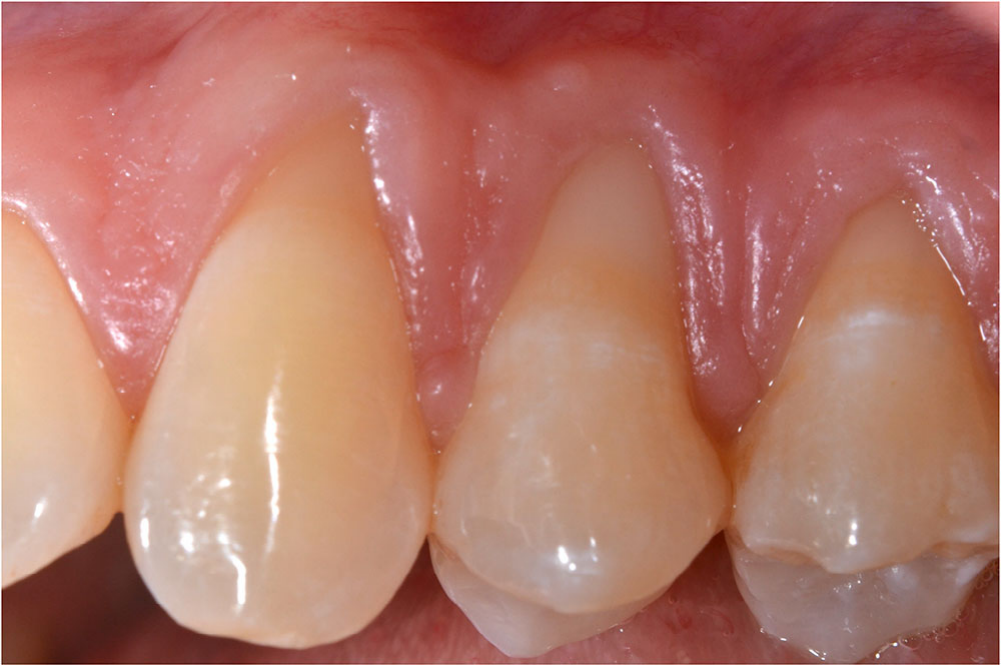
Preoperative image showing multiple recessions in the second quadrant of the upper maxilla.

As in the preoperative picture (Figure 1), a very narrow and scalloped KT apical to recessions of 11 and 12 (1 and 1.5 mm) but wider than 13 (2 mm) can be seen. The most predictable surgical technique chosen in this situation in order to obtain both RC and increase the amount of KT would be the use of a CAF with the adjunct of a connective tissue graft.^10^

It was decided to test a new surgical approach named CAF with an external mucosal release (MR‐CAF) with the objective to cover exposed roots, reduce the tension of the flap, minimize the bleeding, and increase the amount of KT during the healing phase after surgery (1 year).

**FIGURE 2:**
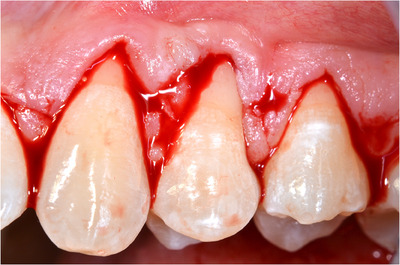
Flap incision as described by Zucchelli and De Sanctis.^9^

**FIGURE 3:**
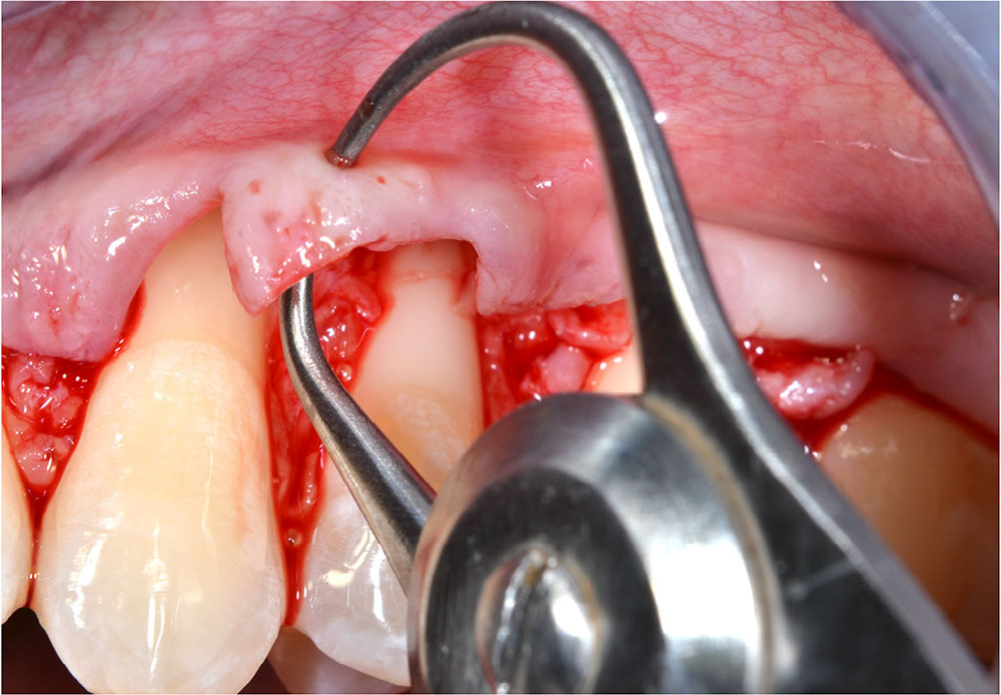
Measurements of the flap with a thickness gauge.

**FIGURE 4:**
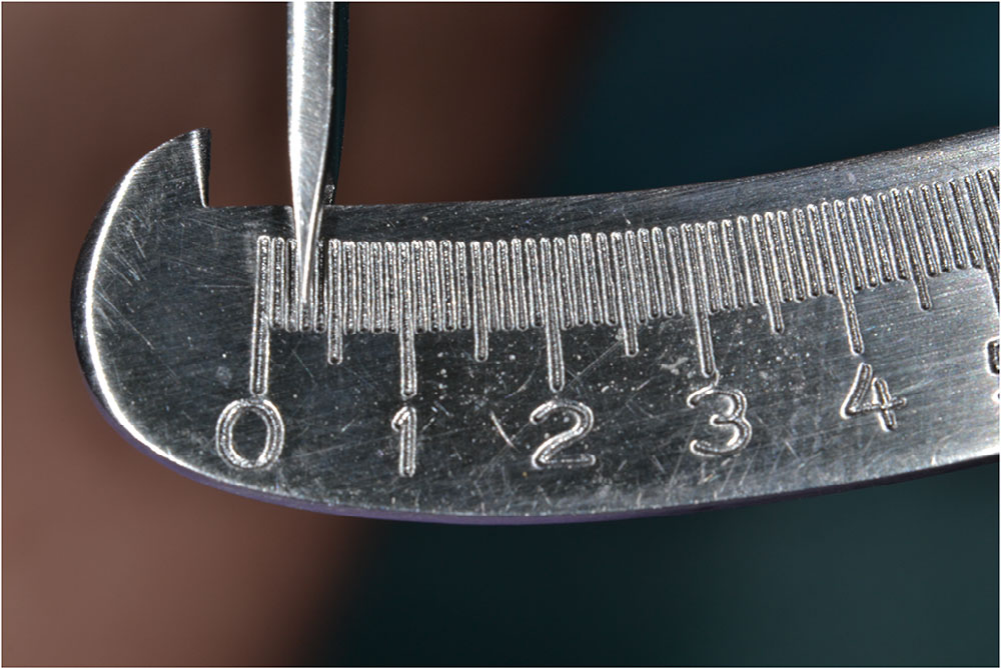
Measurement of flap thickness.

**FIGURE 5:**
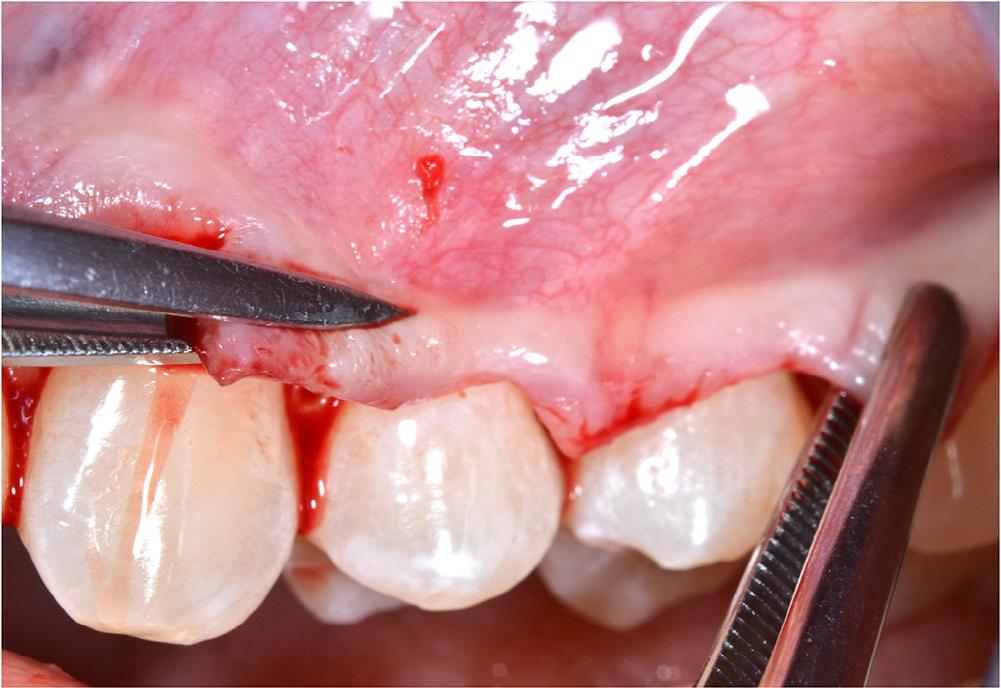
Flap tensioned with two tweezers in order to perform the release incision just above the mucogingival junction.

**FIGURE 6:**
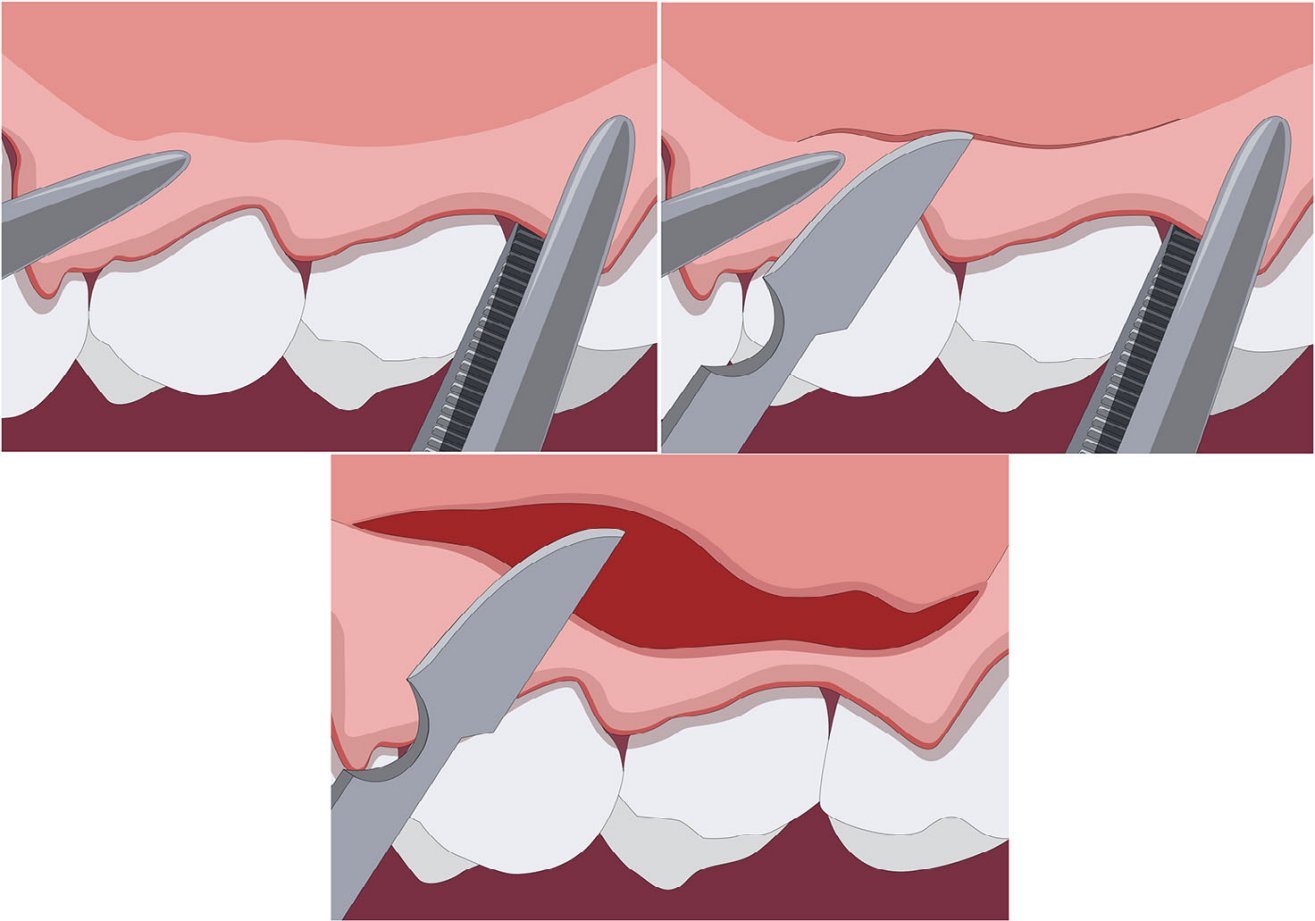
Details of the novel surgical mucosal released coronally advanced flap (MR‐CAF).

**FIGURE 7:**
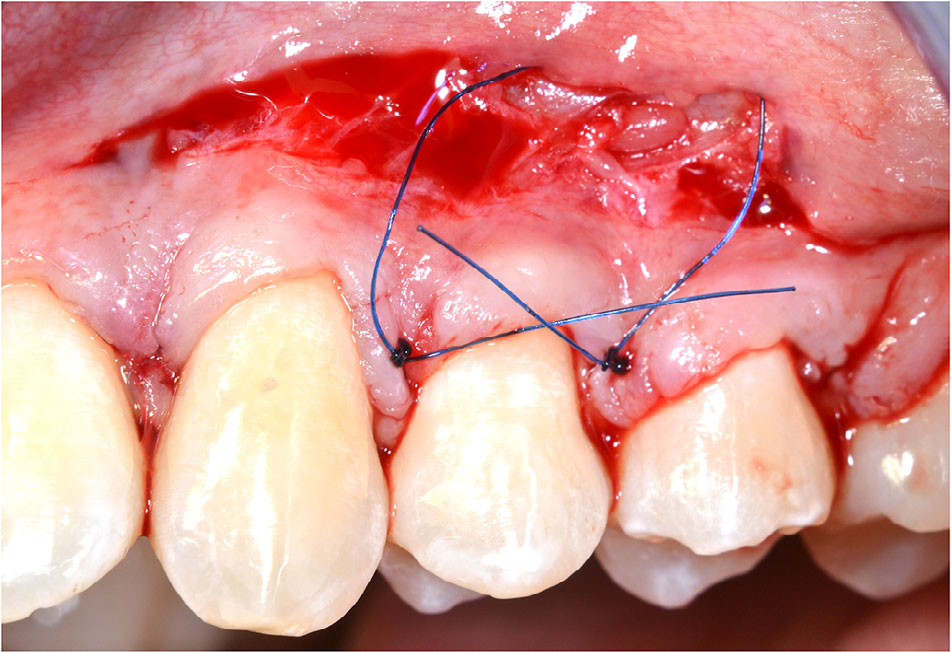
Mucosal release incision and suture.

Under sterile conditions, local anesthesia was given. A CAF as described by Zucchelli and De Sanctis^9^ with the addition of a mucosal release incision was performed by following the surgical steps described below:

‐ Detection of MGJ by means of Lugol's iodine staining.
‐ Intrasulcular incisions involving at least one tooth mesial and at least one tooth distal to the teeth presenting gingival recessions (Figure 2).
‐ Oblique incisions using a split‐thickness approach at the level of the interdental soft tissue in order to elevate each surgical papilla, followed by a full‐thickness flap raised until the MGJ using a periosteal elevator (Figure 2).
‐ Gentle instrumentation of exposed root surfaces and de‐epithelization of the interdental papillae.
‐ The flap thickness was measured with a Boley gauge at the MGJ level (Figures 3 and 4).
‐ Keeping the flap stretched downwards, with two tweezers, an incision was made along the MGJ and deepened for approximately 1 mm, enough to obtain a separation between the oral mucosa and the attached gingiva. The mucosal release incision just above the MGJ was placed slightly beveled undermining the mucosa in order to reduce the risk of fenestrations (Figures 5 and 6).
‐ Passively positioning the split–full–split thickness flap coronally to the CEJ over the involved teeth and stabilizing the flap by means of sling sutures around anatomic papillae (Figure 7).

**FIGURE 8:**
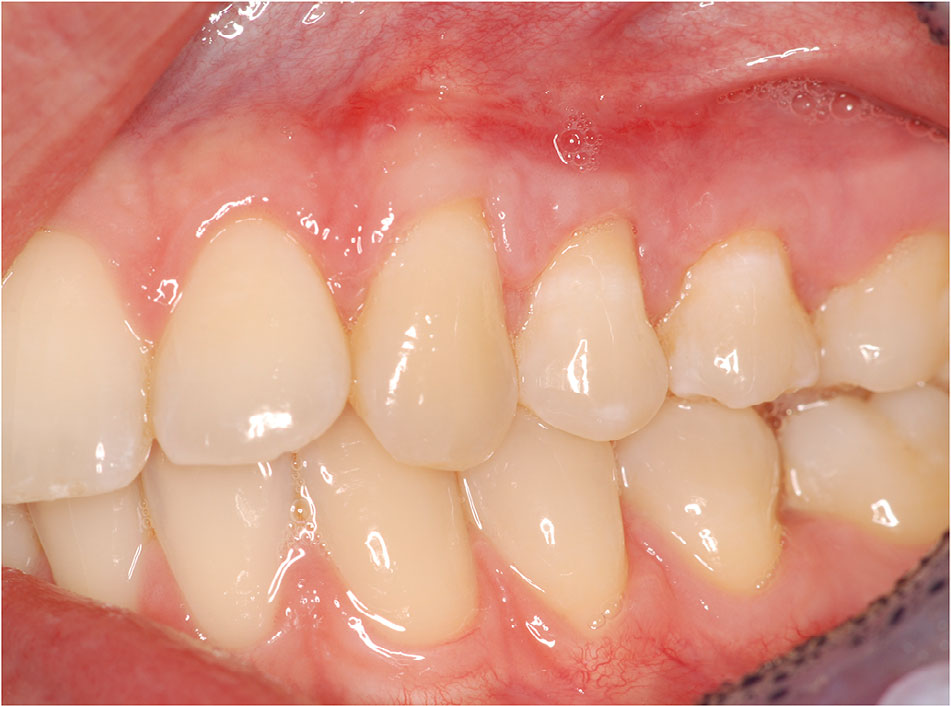
Image showing healing at 15 days following suture removal.

The patient was instructed to refrain from brushing for 2 weeks; 600 mg ibuprofen for postoperative pain and chlorhexidine mouthwash (0.12%) were prescribed. Sutures were removed 2 weeks postoperatively and at the same appointment, a paper‐based visual analog scan (VAS) was administered to measure postoperative pain (Figure 8).

This case report adhered to CARE guidelines.

## RESULTS

### Clinical outcomes

**FIGURE 9:**
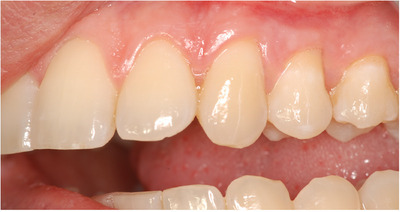
Image showing healing at 1 month following suture removal.

**FIGURE 10:**
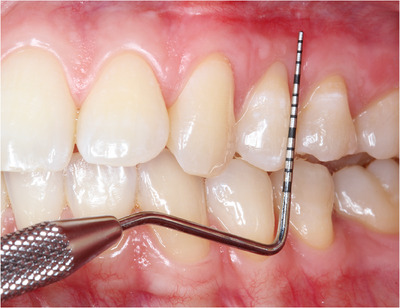
Image showing healing at 6 months following suture removal.

**FIGURE 11:**
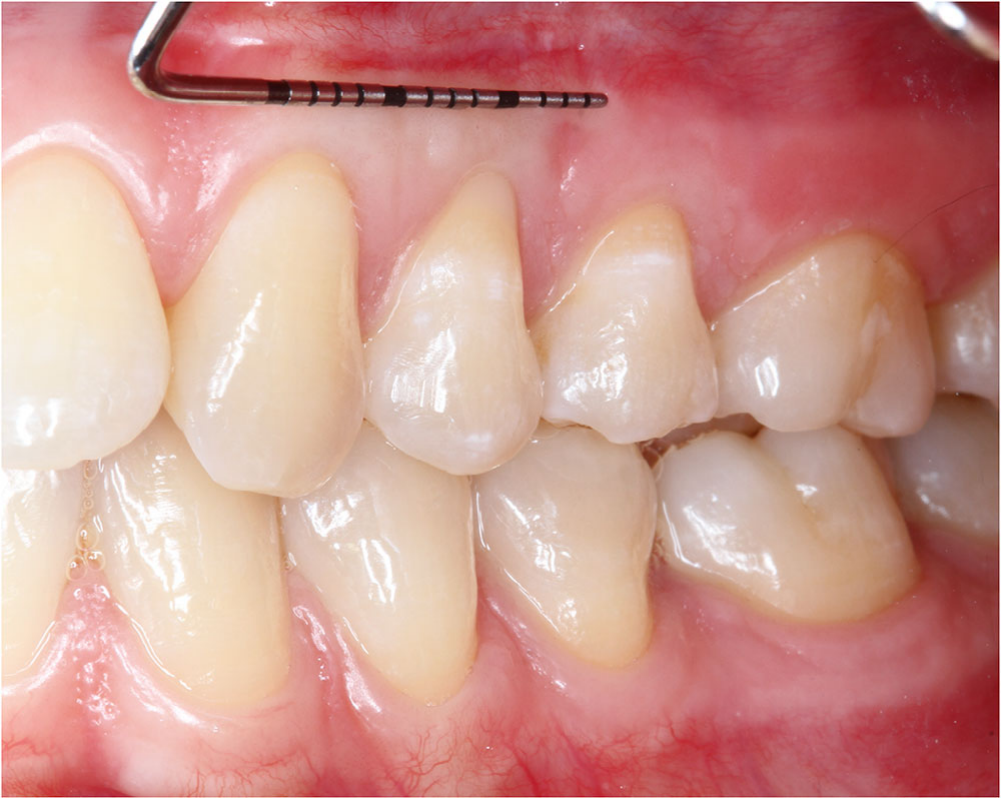
Image showing healing at 1 year following suture removal.

Healing was uneventful. The patient was checked, pictures were taken after 1 and 6 months (Figures 9 and 10), and a final evaluation was done after 1 year (Figure 11). RC at 1 year postoperatively was 95% in tooth 11 and 89% in tooth 12, while CRC was achieved in tooth 13.

The PDs, mid‐buccally on the treated teeth, were limited to 1 mm.

The RC esthetic score for both the clinician and the patient ranged between 9 and 10 with respect to color match and texture.^17^

In Table 1, it is possible to compare all the data of pre‐surgical and post‐surgical situation. One year after the procedure, the results, in terms of RC, soft tissue texture, and color appeared stable, while KT seemed to continue to increase (Figures 10 and 11).

VAS score for postoperative pain was 2.

## DISCUSSION

In this era of patient‐centered esthetic outcomes, restoring the ideal pink and white esthetics is a primary requisite, provided that minimal postoperative discomfort is guaranteed. Therefore, this novel surgical approach seems to fulfill both important patient's demands.

In this case, the average RC achieved was 95% with an increase in KT; this is a strength of our technique, where there is no need to harvest a soft tissue graft with a second surgery. Patient hypersensitivity was also solved and color match and texture could be achieved with no evidence of scar tissue formation in the surgical area.

A releasing incision was performed to improve passive release of the flap. It is placed along the MGJ that is best visualized with the application of Lugol's iodine staining over the area.

The strength of the MR‐CAF technique is to guarantee maximum flap vascularity and reduction of patient's discomfort and reduction in pain killer prescription by performing a tension‐releasing incision predominantly on the external side, with less damage to blood vessels and muscles.

A reduction in the external tension of the mucosa allows the coronal advancement of the flap, with proper positioning over the exposed roots.

The possible mechanisms underlying the increase in KT can stem from cellular and biological reasons. With the mucosal incision, right at the level of the MGJ, a second intention healing is created over an area that has shown to be characterized by a specific pattern of wound healing with a high expression of fibrotic markers^20^ and autophagy activation, demonstrating a higher soft tissue contraction with increased production of myofibroblasts, which is typical of “scarring” healing. On the contrary, when the incision is placed over attached gingiva, the expression of genes toward a scarring pattern is reduced along with reduced differentiation toward the myofibroblastic lineage involved in the healing process.^21^ This latter cellular event might explain the possible “creeping” of keratinocytes into the wounded area leading to an increase in KT as a result of the secondary healing process.

One important aspect when selecting this surgical method is regarding the necessary operator skills. Experienced clinicians with the use of magnifying lenses are to be considered as the mandatory prerequisites in order to best perform the releasing incision along the MGJ.

## CONCLUSIONS

In conclusion, the results of the present study demonstrated that this new approach involving CAF with the addition of an external releasing incision for the treatment of multiple gingival recessions in patient with esthetic demands, although requiring necessary operator skills, seems to offer an effective clinical outcome in terms of RC and an increase in KT but with minimal patient's postoperative discomfort, as underlined by the VAS score. The MR‐CAF is a promising technique where the CAF technique alone may not be an indication.

## AUTHOR CONTRIBUTIONS

*Conceptualization (lead), review and editing (equal), and visualization (lead)*: Andrea Pilloni. *Writing—original draft (lead), review and editing (equal), and visualization (supporting)*: Fabiola Dell'Olmo.

## CONFLICT OF INTEREST STATEMENT

The authors declare they have no conflicts of interest.
